# Assessment of Levels of Apelinergic System Peptides in Serum and Epicardial Adipose Tissue in Patients with Multivessel Coronary Artery Disease Who Underwent Myocardial Revascularisation

**DOI:** 10.3390/biomedicines13040809

**Published:** 2025-03-27

**Authors:** Maciej Rachwalik, Anna Leśków, Małgorzata Matusiewicz, Agnieszka Jama-Kmiecik, Dorota Diakowska

**Affiliations:** 1 Department of Cardiac Surgery and Heart Transplantation, Institute of Heart Diseases, Wroclaw Medical University, Borowska 213 Street, 50-556 Wroclaw, Poland; 2 Division of Medical Biology, Faculty of Nursing and Midwifery, Wroclaw Medical University, Chałubińskiego 3 Street, 50-368 Wroclaw, Poland; agnieszka.jama-kmiecik@umw.edu.pl; 3 Division of Medical Biochemistry, Department of Biochemistry and Immunochemistry, Wroclaw Medical University, Chałubińskiego 10 Street, 50-368 Wrocław, Poland; malgorzata.matusiewicz@umw.edu.pl

**Keywords:** atherosclerosis, coronary artery disease, elabela, apelin, apelin receptor

## Abstract

**Background:** Peptides of the apelinergic system may participate in the development of atherosclerosis, but their role in atherogenesis is unclear. The aim of the study was to evaluate the levels of apelinergic system peptides, such as Elabela (Ela), apelin-13 (AP-13), apelin-17 (AP-17) and apelin receptor (APJ) in the serum and epicardial adipose tissue (EAT) of patients with multivessel coronary artery disease (CAD) who underwent myocardial revascularisation surgery. **Methods:** The participants comprised 51 CAD patients and 34 healthy adults. Concentrations of Ela, AP-13, AP-17 and APJ were determined by ELISA kits. We analysed the demographics, and clinical and laboratory parameters of the CAD patients. **Results:** We showed that the serum Ela and AP-17 levels significantly decreased, and APJ significantly increased, in the CAD patients in comparison to the healthy control. A significant relationship between the serum and EAT concentrations of Ela and APJ (*p* < 0.05) was observed. Positive correlations were found between the serum levels of AP-13 and AP-17, and between AP-17 and APJ. There was a positive correlation between the tissue levels of AP-17 and APJ. The tissue Ela concentration negatively correlated with the BMI, TCH and LDL levels. AP-13 in EAT was negatively associated with the glucose level. In contrast, the tissue APJ showed a positive correlation with TCH concentration. Good diagnostic potential of ELA, AP-17 and APJ was observed for CAD prediction (*p* < 0.001 for all). **Conclusions:** The results indicate that the levels of apelinergic peptides are altered in patients with CAD, which may be a potential diagnostic indicator.

## 1. Introduction

Atherosclerosis is a chronic inflammatory disease of the endothelium and arterial intima in which a disturbance of the balance between pro-inflammatory and inflammation-resolving mechanisms results in development of arterial stiffness [1,2]. It is assumed that the apelin (AP) and Elabela (Ela) peptides included in the apelinergic system play a role as mediators of the endothelial function and atherosclerosis [2,3].

The apelinergic system consists of the following peptides: a G-protein-coupled receptor (APJ receptor) and its two endogenous ligands Ela and AP. Various circulating isoforms of both Ela and AP ligands have been identified, which differ in the amino acid sequence in the peptide chain [2,3]. AP and Ela are expressed by cells of various organs, such as the adipose tissue, heart, lung, kidney, gastrointestinal tract, liver and central nervous system [2,3,4]. Expression of the APJ receptor was detected in cardiomyocytes, fraction of fibroblasts, endothelial cells and vascular smooth muscle cells in a variety of vessels [2,4].

Apelinergic system peptides exert an opposite effect on the renin–angiotensin system and influence its regulation [2,5]. As shown in experimental study, blocking the angiotensin II-type 1 receptor (AT1) increases AP and APJ receptor expression, which results in a protective effect on the heart muscle [5]. Inversely, the infusing of angiotensin II down-regulates the expression of cardiac apelin mRNA, which induces myocardial remodelling and the development of aortic aneurysm [2,5,6].

The Ela–APJ axis suppresses angiotensin II-induced hypertension, cardiac hypertrophy and fibrosis [7]. It has been shown that Ela has a small effect on ACE2 expression in the cardiomyocytes; however, it down-regulates ACE expression, antagonizing the effect of angiotensin II in the heart [4,7].

Clinical studies in healthy volunteers confirm that apelinergic system peptides are protective against hypertension [2]. Apelin promotes nitric oxide synthesis, inducing vasodilatation, and Elabela causes direct vasorelaxation of vascular smooth muscle cells [2,4,7]. Elabela attenuates the effects of the increase in blood pressure induced by angiotensin II [2,4].

Visceral, especially periadventitial, adipose tissue is considered a very active fat depot, with an established role in vascular physiology and atherosclerotic processes [2,4,8]. The role of AP/APJ in the pathogenesis of atherosclerosis has not been fully defined. Experimental studies have shown that apelin promotes vascular smooth muscle cell proliferation, and a decrease in APJ protects against atherosclerotic plaque formation [2,9]. In another study, a loss of AP promoted atherosclerosis, and treatment with AP decreased angiotensin II-induced atherosclerosis [10]. In humans, AP and APJ expression was reduced in aortic vascular smooth muscle cells during atherosclerosis, which may account for increased plaque vulnerability [8]. The importance of Ela in the development of atherosclerosis is still unclear. Recently, the role of adipokines in pericoronary epicardial adipose tissue [EAT] has been of particular interest [8,11]. EAT has been shown to have a high activity of pro-inflammatory factors, including adipokines, which may influence the formation of atherosclerotic plaque in the coronary arteries [12,13]. Therefore, analysis of the levels of apelinergic system peptides in EAT may be a good indicator of the risk of developing atherosclerosis.

The aim of this study was to evaluate the levels of apelinergic system peptides, such as Ela, AP-13, AP-17 and APJ, in the serum and epicardial adipose tissue in patients with multivessel coronary artery disease who underwent myocardial revascularisation surgery.

## 2. Materials and Methods

### 2.1. Study Design and Participants

The Bioethics Committee at Wroclaw Medical University approved the study (approval code no. KB-254/2024, approval date 26 April 2024), and each patient signed informed consent to participate.

The study group consisted of 51 patients referred for elective coronary artery bypass grafting (CABG) due to advanced CAD which was carried out in the Clinical Department of Cardiac Surgery and Heart Transplantation, Institute of Heart Diseases, University Clinic Hospital (Wroclaw, Poland) in 2024.

All participants underwent myocardial revascularisation with the use of extracorporeal circulation. The average hospital stay for these patients was 6 days, with a standard deviation of 3 days. Baseline clinical data were collected for all participants.

The study cohort was randomly selected from a total pool of 210 patients who underwent cardiac surgery at the clinic during the study period, which spanned from May 2024 to October 2024. To be included, patients were required to meet specific criteria.

#### Inclusion and Exclusion Criteria

Eligible participants had triple-vessel coronary artery disease (3VD), were in sinus rhythm according to their electrocardiograms (ECGs), were under the age of 80, and had been receiving statin therapy prior to surgery. Participants also provided written consent to join the study. Triple-vessel disease is indicative of advanced atherosclerosis, where coronary artery damage is often associated with hypercholesterolemia and other contributing factors affecting epicardial tissue. It was hypothesised that these patients would exhibit elevated apelinergic system peptide levels and higher-quality epicardial tissue homogenates compared to individuals with less severe CAD.

The participants were already under treatment for CAD, which included medications such as antihypertensive drugs, beta-blockers, aspirin and statins. The study aimed to maintain consistency by including patients with similar treatment regimens.

Exclusion criteria included moderate or severe valvular heart disease necessitating additional surgical intervention, left ventricular ejection fraction (LVEF) below 30%, insulin-dependent diabetes (as defined by the Polish Diabetology Society guidelines), end-stage renal disease, rheumatic diseases, infections or cancer.

As a reference, sera from 34 blood donors, obtained from the Regional Center of Transfusion Medicine and Blood Bank, Wroclaw, Poland, were used. All blood donors were considered healthy on the basis of physical examination and routine blood tests, the results of which indicated that they were within the reference range.

Demographic characteristics of the CAD patients and the control group are presented in Table 1. The control group consisted of 27 (79.41%) men and 7 (20.59%) women, with a mean age of 61.67 ± 1.55 years old. In the group of CAD patients, there were 40 (78.43%) men and 11 (21.56%) women, with a mean age of 63.74 ± 7.15 years old. There were insignificant differences in the sex distribution (*p* = 0.831) and mean age (*p* = 0.101) between the CAD patients and the control group.

### 2.2. Measurement of Serum and Tissue Levels of Apelinergic System Peptides

Data on the haematological variables, glucose, glycated haemoglobin HbA1c, total cholesterol (TCH), HDL and LDL cholesterol, triglycerides (TG) and C-reactive protein (CRP) were retrieved from the patients’ medical records.

For the analysis of the apelinergic system peptides, venous blood samples (2 mL) were collected from the CAD patients with the use of a vacuum system, before surgery, after overnight fasting, at least 24 h after admission, for routine laboratory tests. The blood samples were collected into tubes with separating gel (Beckton Dickinson, Plymouth, UK), clotted for 30 min. at room temperature, then centrifuged at 3000× *g* (MPW 260R, MPW Med. Instruments, Warsaw, Poland) for 15 min at room temperature. The obtained sera were stored at −20 °C.

Then, 51 tissue samples of epicardial adipose tissue (EAT) were collected intraoperatively from the same patients and frozen at −80 °C until further analysis. A FastPrep homogeniser (MP Biomedicals, Santa Ana, CA, USA) was used to homogenise the EAT samples, and then PBS buffer (Sigma-Aldrich, St. Luis, MO, USA) containing PMSF (Sigma-Aldrich, St. Luis, MO, USA) was used in the next steps. Centrifugation was performed twice for 5 min at 14,000× *g* and 4 °C. The second centrifugation was performed just before the analysis.

The concentrations of ELA, AP-13, AP-17 and APJ receptors in the serum and EAT samples were measured by ELISA kits (Shanghai Sunred Biological Technology Co., Ltd., Shanghai, China). The ELISA experiments were conducted in duplicate. All tests were performed according to the manufacturer’s instructions. The sensitivity of the ELA assay was 13.7 pg/mL. The sensitivity of the APJ receptor was 0.288 ng/mL, while the intra- and inter-assay CVs were <10.0% and <12.0%, respectively. The sensitivity of the AP-13 assay was 0.668 pg/mL, and the intra- and inter-assay CVs were <10.0% and <12.0%, respectively. The minimum detectable dose of AP- 17 was 0.674 pg/mL, and the intra- and inter-assay CVs were <10.0% and <12.0%, respectively.

### 2.3. Statistical Analysis

The distribution of the data was tested with the Shapiro–Wilk normality test. Depending on the type of distribution, descriptive data for quantitative variables were shown as mean and standard deviation (±SD) or the median and interquartile range (IQR Q1–Q3). Descriptive data for qualitative data were presented as the number and percentages. The results obtained for apelinergic system peptides showed a not-normal distribution; therefore, nonparametric tests were used for the statistical analyses. The Pearson chi-square test or the Mann–Whitney test were used to analyse the differences between two independent groups. Dependent samples were compared using the Wilcoxon test. Correlations were analysed using the Spearman’s rank correlation test. The diagnostic utility of the selected peptides of apelinergic system was performed by Receiver Operating Characteristic (ROC) analysis. All values of *p* < 0.05 were assumed to be statistically significant. Data were analysed using Statistica v. 13.3 (Tibco Software Inc., Palo Alto, CA, USA).

## 3. Results

### 3.1. Characteristic of CAD Patients

The baseline characteristics of the CAD patients are shown in Table 2. The mean BMI index of the patients was above the norm (28.00 ± 3.90), five of them (9.80%) had diabetes and five people (9.80%) had suffered a myocardial infarction. An increased concentration of glycated haemoglobin HbA1c was observed (6.44 ± 1.19%). In the group of CAD patients, heart failure (HF) was observed in eleven (21.57%) subjects: eight patients with mildly reduced ejection fraction (HFmrEF) and three patients with reduced ejection fraction (HFrEF).

### 3.2. Serum and Tissue Levels of Apelinergic System Peptides

**Figure 1:**
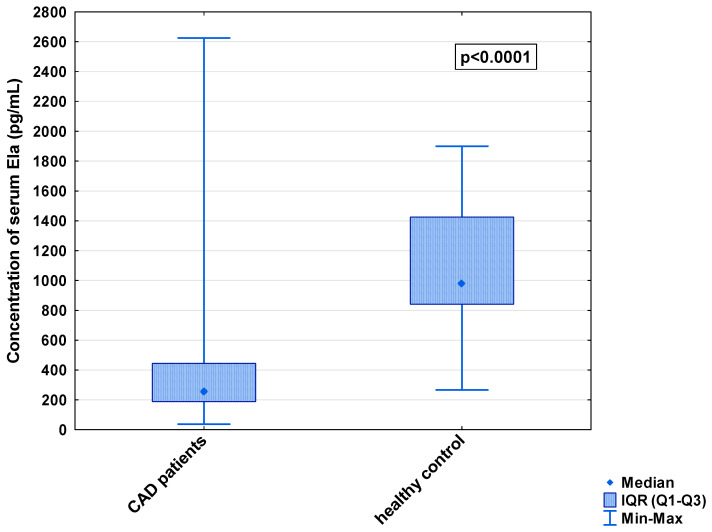
Serum concentration of Ela in CAD patients and healthy controls.

**Figure 2:**
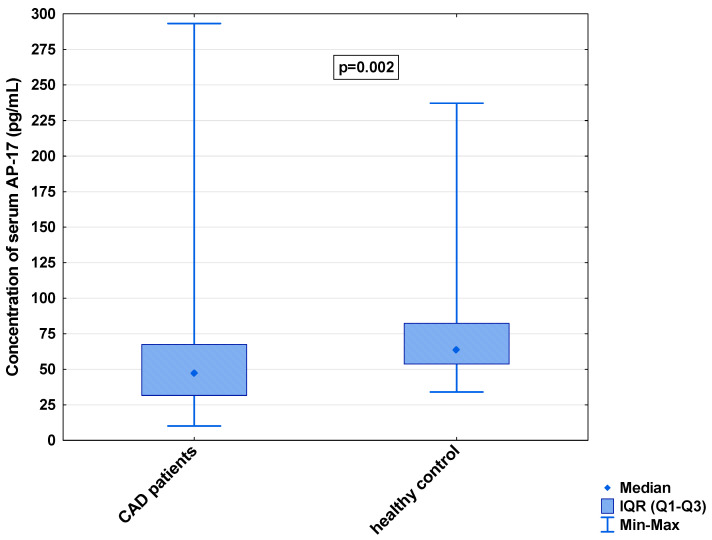
Serum concentration of AP-17 in CAD patients and healthy controls.

**Figure 3:**
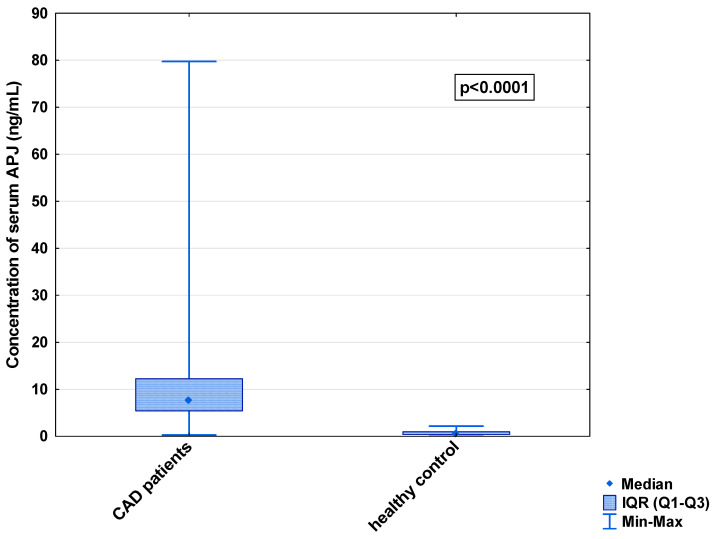
Serum concentration of APJ in CAD patients and healthy controls.

**Figure 4:**
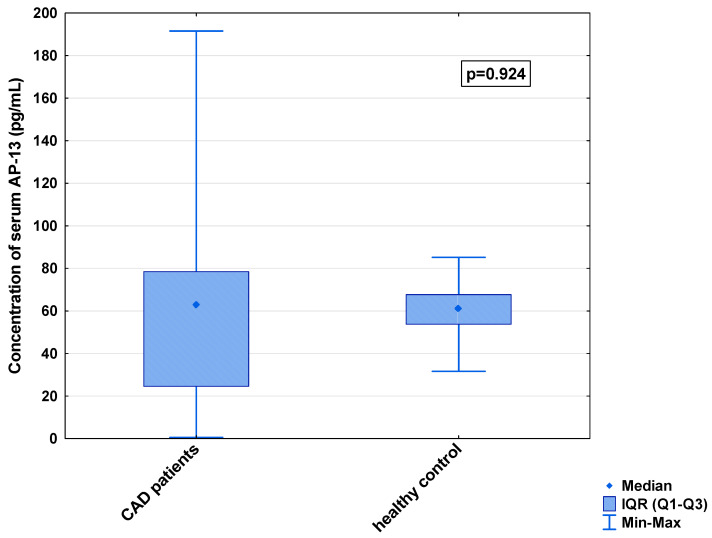
Serum concentration of AP-13 in CAD patients and healthy controls.

In the serum, a decrease in the median concentration of Ela (252.00 [IQR: 188.75–445.00] pg/mL vs. 979.16 [IQR: 841.66–1425.00] pg/mL) and a decrease in the median concentration of AP-17 (47.16 [IQR: 31.60–67.50] pg/mL vs. 63.75 [IQR: 53.74–82.25] pg/mL], but an increase in the median level of the APJ receptor (7.65 [IQR: 5.43–12.27] ng/mL vs. 0.48 [IQR: 0.39–0.99] ng/mL), were found in the CAD patients in comparison to the healthy controls (*p* < 0.001 for all) (Figure 1, Figure 2 and Figure 3). An insignificant difference was also observed in the median concentration of AP-13 between the CAD patients and control groups (62.70 [IQR: 24.60–78.46] pg/mL vs. 61.23 [IQR: 53.80–67.66] pg/mL) (Figure 4).

**Figure 5:**
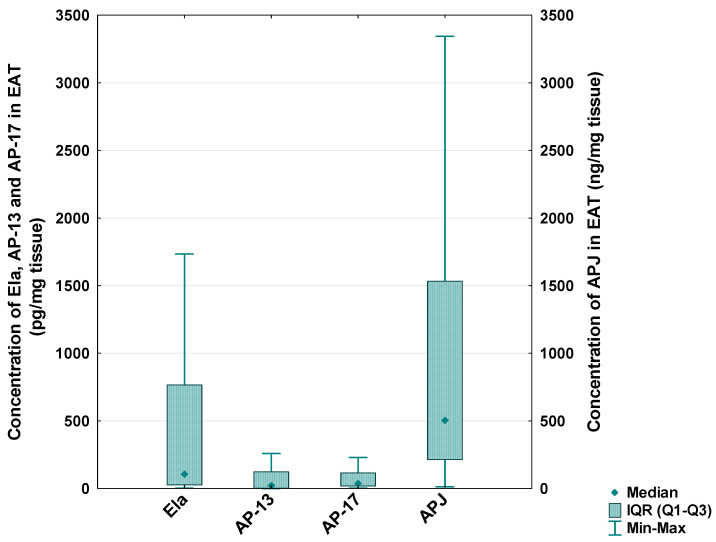
Concentration of apelinergic system peptides in epicardial adipose tissue (EAT) of CAD patients.

In the CAD patients, the median level of Ela in the EAT was 105.73 [IQR: 26.80–776.10] pg/mg tissue, the median concentration of AP-13 was 23.58 [IQR: 3.26–123.38] pg/mg tissue, the median concentration of AP-17 was 40.00 [IQR: 18.17–115.04] pg/mg tissue and the median level of the APJ receptor was 505.78 [IQR: 213.59–1533.68] ng/mg tissue (Figure 5).

A pairwise comparison of the concentrations of the apelinergic system peptides showed a significant relationship between EAT and the serum levels of Ela and APJ receptor (*p* < 0.05 for both) (Table 3).

### 3.3. Correlations Between Selected Study Parameters in CAD Patients

**Figure 6:**
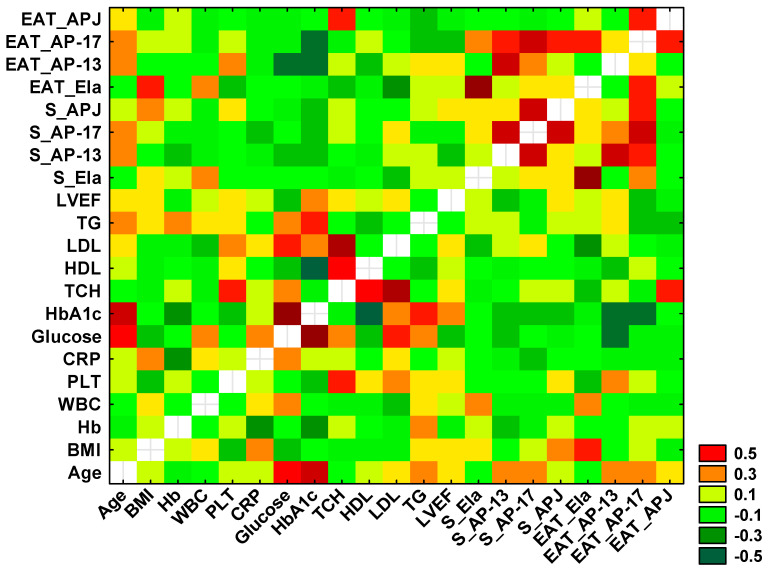
Heatmap for correlation coefficients of selected variables. Orange, red and dark green indicate statistically significant correlations between tested parameters.

Using Spearman’s rank correlation test, statistically significant positive and negative correlations were found between selected variables, which are presented in the heat diagram in Figure 6.

A significant positive correlation between the concentration of serum and tissue Ela (r = 0.758, *p* < 0.0001) was observed, as well as a positive association between the serum levels of AP-13 and AP-17 (r = 0.553, *p* < 0.0001), between the serum and tissue AP-13 (r = 0.539, *p* < 0.001) and between the serum AP-13 and tissue AP-17 (r = 0.344, *p* = 0.013).

A positive correlation was also demonstrated between the serum AP-17 and serum APJ (r = 0.540, *p* < 0.0001) and between the serum and tissue AP-17 (r = 0.538, *p* < 0.0001). The serum concentration of APJ was correlated with the tissue AP-17 (r = 0.307, *p* = 0.028), and the tissue APJ was associated with the tissue AP-17 (r = 0.311, *p* = 0.026).

Significant negative correlations were found between the concentration of tissue Ela and serum TCH (r = −0.293, *p* = 0.039), LDL (r = −0.337, *p* = 0.016) and BMI (r = −0.305, *p* = 0.030).

Moreover, a significant negative association between the tissue AP-13 concentration and serum glucose (r = −0.424, *p* = 0.038) and a positive correlation between the tissue APJ and serum TCH (r = 0.345, *p* = 0.014) were shown.

Finally, a positive correlation between the serum level of glucose and HbA1c (r = 0.721, *p* = 0.003) was observed.

### 3.4. Diagnostic Potential of Serum Levels of Ela, AP-17 and APJ in CAD Patients

Significant differences between the study and healthy control groups indicated that reduced Ela or AP-17 and increased APJ levels may be considered as risk factors for CAD development.

**Figure 7:**
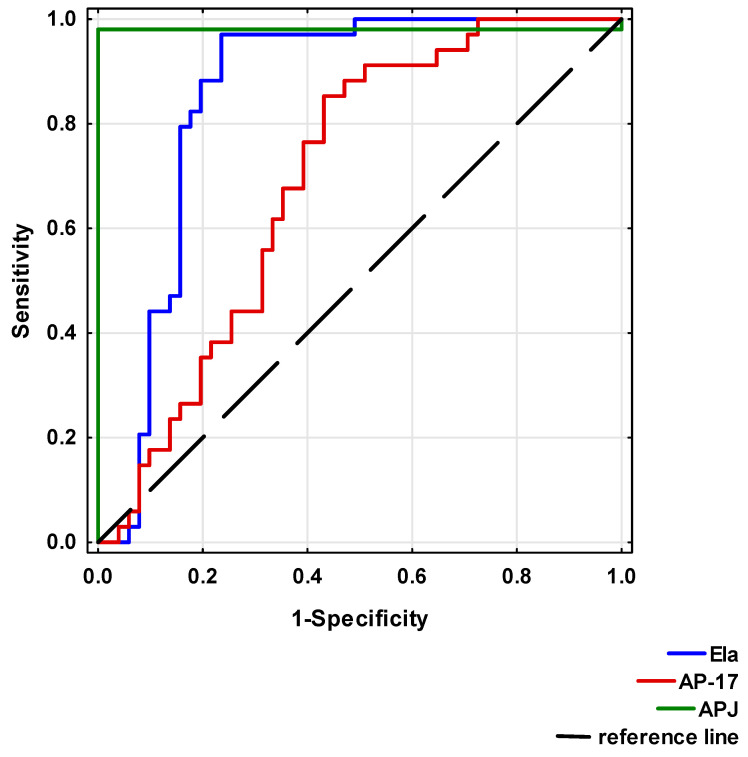
Receiver Operating Characteristic (ROC) curves of Ela, AP-17 and APJ for CAD diagnosis.

To analyse the diagnostic value of Ela, AP-17 and APJ, ROC curves were calculated (Table 4 and Figure 7). A circulating Ela concentration at a cut-off point below 495.83 pg/mL, a serum AP-17 concentration at a cut-off point below 49.50 pg/mL and a serum APJ level at a cut-off point above 2.83 ng/mL may potentially present prognostic values for a CAD diagnosis.

## 4. Discussion

The role of peptides of the apelinergic system in the pathogenesis of atherosclerosis has not been fully defined. However, many studies have reported that the activity of apelin or Elabela may exert a beneficial effect on patients with coronary artery disease due to their vasodilatory effects on coronary arteries and their cardioprotective potential [2,3,6,7,10,11,14].

Our data have demonstrated that the serum Ela concentration was significantly reduced and correlated with EAT in CAD patients who underwent myocardial revascularisation. Also, the serum AP-17 concentration significantly decreased in the CAD patients. In contrast, the serum APJ concentration was significantly elevated, and a relationship was observed between the levels of APJ in the serum and EAT. The ROC analysis showed that Ela, AP-17 and APJ may have potential as biomarkers in CAD diagnosis.

These data agree with our previous study, where we observed reduced serum levels of Ela and AP-17 in patients with chronic coronary syndrome than in patients after myocardial infarction and in the control group. In contrast, concentration of APJ was higher in patients with chronic coronary syndrome compared to controls or acute coronary syndrome patients [15].

Our results are also consistent with previous studies that found a decrease in apelin levels in CAD patients compared to the controls [16,17]. A decrease in apelin concentrations was independently associated with CAD severity and acute coronary syndrome incidence [16,17]. Liu et al. [18] reported that apelin-13 induces cellular cholesterol efflux from macrophage-derived foam cells and reduces their formation, which indicates a potential antiatherogenic function of this peptide. Other experimental studies showed that apelin can stimulate NO production, which promotes the inhibition of angiotensin II cellular signalling [10,19].

Li et al. [20] demonstrated a decrease in the concentration of circulating Ela in hypertensive patients. They suggest that this reduction in Ela synthesis may be involved in the pathogenesis of hypertension-related vascular dysfunction. Also, the study by Tian et al. [21] confirmed that serum Ela levels decreased in patients with hypertension, especially malignant hypertension, and this peptide is a potential marker of hypertension-related renal damage. The results of a meta-analysis study showed that lowering the circulating apelin level was significantly associated with an increased risk of hypertension [22].

The above results indicate that apelinergic system peptides exert an antiatherogenic effect on the vascular vessels and reduce the progression of atherosclerosis.

In the present study significant positive correlations were observed between serum AP-13 and AP-17, serum AP-17 and APJ and tissue AP-17 and APJ. These results confirmed that peptides of the apelinergic system are significantly interrelated, influencing the regulation of circulatory system functions [2,3,4,14].

Increased BMI and increased serum TCH and LDL levels were significantly associated with decreased Ela expression in epicardial adipose tissue. To our knowledge, this is the first study to analyse the relationship between the serum or EAT concentration of Ela and the lipid profile. We suggest that a decrease in Ela production in epicardial adipose tissue might have an influence on the high level of serum TCH and LDL. In previous experimental studies, apelin was shown to be an inhibitor of adipogenesis and lipolysis and the factor that stabilised lipid vacuoles by making them resistant to lipases [23,24].

In addition, the study by Riazian et al. [25] showed an inverse correlation between apelin and BMI, which suggested that pathologic conditions such as CAD might have an effect on the serum levels of apelin compared to BMI and adipose tissue.

Higher levels of serum glucose were significantly correlated with decreased AP-13 expression in the EAT. In an earlier study, the authors suggest that the apelin level is related to enhanced serum lipids and can be used as a predictor of atherosclerosis in diabetic patients [26].

A significant positive correlation was observed between the serum TCH levels and APJ expression in adipose tissue. A previous meta-analysis study reported that the apelin/HDL-c, apelin/LDL-c and apelin/TCH ratios could be used as diagnostic markers for cardiovascular diseases [26]. Our results indicate the presence of a relationship between the parameters of carbohydrate and lipid metabolism and the expression of apelinergic system peptides in the EAT.

### Limitations of the Study

The main limitation of our study is the small sample size, which stems from significant reasons. The process of obtaining material from epicardial tissue is complex and requires the fulfilment of very stringent clinical and technical criteria. The patient group was carefully selected based on age, ejection fraction, absence of significant comorbidities and clinical stability. Patients meeting these criteria are rarely encountered in cardiac surgery practice as the majority are critically ill and require emergency intervention. An additional limiting factor, while also ensuring maximum homogeneity of the sample, was the collection of material by a single cardiac surgeon, which helped to avoid errors during sample collection, preservation and storage prior to measurements. In the future, we plan to expand the study to include a larger number of patients.

The majority of the CAD patients were treated with statins—42 subjects (82.35%)—which is an integral part of cardiovascular diseases prevention. This experimental study demonstrated that apelin/APJ signalling is an important regulator of statin effects in vascular endothelial cells [27]. A decrease in apelin expression was connected with inhibition of the positive effect of statins on endothelial homeostasis parameters, and statins can induce APJ expression [27]. The results of our study showed an increase in the APJ concentration in serum of CAD patients, which could have been influenced by the statins treatment.

## 5. Conclusions

Our findings demonstrate that the levels of ELA, AP-13, AP-17 and APJ are altered in patients with CAD. The studied parameters show potential as biochemical indicators of changes in patients with CAD, which should be investigated in the future on a larger sample of patients.

## Figures and Tables

**Table 1:** Demographic characteristic of CAD patients and healthy controls. Descriptive data are presented as number (percentage) or mean (±SD).

Variable	Healthy Control (n = 34)	CAD Patients (n = 51)	*p*-Value
Sex			0.831
Male	27 (79.41)	40 (78.4)	
Female	7 (20.59)	11 (21.56)	
Age (years)	61.67 ± 1.55	63.74 ± 7.15	0.101

**Table 2:** CAD patients’ characteristics.

Variable	CAD Patients (n = 51) [Mean ± SD or Median [IQR] ^#^ or Number (%)]
Height (cm)	171.10 ± 0.07
Weight (kg)	82.34 ± 14.75
BMI (kg/m^2^)	28.00 ± 3.90
BMI:	
Norm (<24.99)	11 (21.56)
Overweight (25.00–29.99)	26 (50.98)
Obese (>30)	14 (27.45)
Diabetes mellitus	5 (9.80)
LVEF (%)	55.53 ± 8.34
Heart Failure Phenotype:	
HFpEF	40 (78.43)
HFmrEF	8 (15.69)
HFrEF	3 (5.88)
Myocardial infarction	5 (9.80)
Stents	4 (7.84)
Statins	42 (82.35)
Haemoglobin (g/dL)	13.49 ± 2.61
WBC	7.54 ± 3.02
PLT	230.26 ± 75.32
CRP (mg/L)	1.77 [0.94–5.63] ^#^
Glucose (mg/mL)	100.00 [93.00–115.50] ^#^
HbA1c (%)	6.44 ± 1.19
TCH (mg/mL)	166.58 ± 37.48
HDL (mg/mL)	56.24 ± 38.54
LDL (mg/mL)	87.76 ± 34.85
TG (mg/mL)	124.83 ± 48.78

BMI: body mass index; CRP: C-reactive protein; HFpEF: heart failure with preserved ejection fraction; HFmrEF: heart failure with mildly reduced ejection fraction; HFrEF: heart failure with reduced ejection fraction; HbA1c: glycated haemoglobin; LVEF: left ventricular ejection fraction; TCH: total cholesterol; TG: triglycerides. # IQR values are in square brackets

**Table 3:** Pairwise comparison of concentrations of Ela, AP-13, AP-17 and APJ in EAT and serum of CAD patients. Descriptive data were presented as medians [IQR].

Parameter	EAT Sample	Serum Sample	*p*-Value
Ela	105.73 pg/mg tissue [26.80–766.10]	252.00 pg/mL [188.75–445.00]	0.012 *
AP-13	23.58 pg/mg tissue [3.26–123.38]	62.70 pg/mL [24.60–78.46]	0.575
AP-17	40.00 pg/mg tissue [18.17–115.04]	47.16 pg/mL [31.60–67.50]	0.779
APJ	505.78 ng/mg tissue [213.59–1533.68]	7.65 ng/mL [5.43–12.27]	<0.0001 *

*: statistically significant; EAT: epicardial adipose tissue.

**Table 4:** Diagnostic potential of serum Ela, AP-17 and APJ in CAD patients.

	Ela	AP-17	APJ
AUC (95%CI)	0.855 (0.769–0.941)	0.696 (0.586–0.807)	0.980 (0.942–1.00)
*p*-value	<0.0001 *	<0.001 *	<0.0001 *
Cut-off point	495.83 pg/mL	49.50 pg/mL	2.83 ng/mL
Sensitivity	0.971	0.853	0.961
Specificity	0.765	0.569	0.971

*: statistically significant; AUC: area under the ROC curve.

## Data Availability

The data are available from corresponding author and may be shared if necessary.
